# Supplementary material for: A CNOT gate between multiphoton qubits encoded in two cavities
Source: Nat Commun. 2018 Feb 13;9:652. doi: 10.1038/s41467-018-03059-5 (PMC5811561; doi:10.1038/s41467-018-03059-5)
Supplement: Supplementary file 1 — Supplementary Information [file 41467_2018_3059_MOESM1_ESM.pdf]

Supplementary Note 1: Experimental device

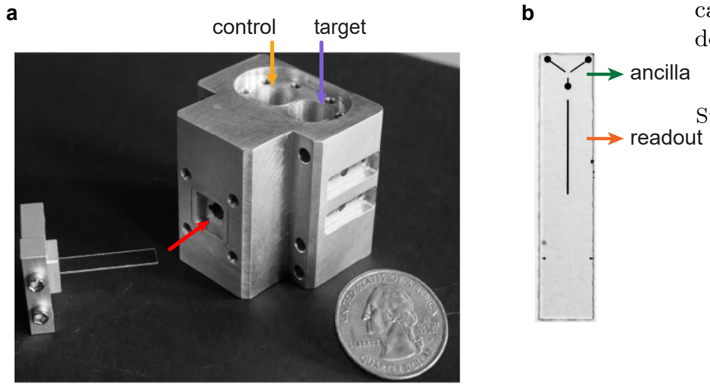

Supplementary Figure 1: **Overview of the experimental device. a,** Photograph of the aluminum package housing the control and target cavities. The sapphire chip containing an ancilla transmon and a stripline readout resonator is clamped by an aluminum holder and inserted into a designated tunnel in the package. **b,** Micrograph of the sapphire chip.

The experimental device consists of a 3D structure made from high-purity aluminum, containing two high-Q coaxial stub cavities[1], as well as a tunnel in which we insert a sapphire chip containing a fixed-frequency ancilla transmon and a stripline readout resonator[2] (Supplementary Figure 1a). The fundamental modes of the superconducting cavities are used for encoding the control and target qubits[3]. The ancilla (with anharmonicity of $2\pi\times117$ MHz) has three antenna pads providing coupling to both cavities and to the readout resonator (Supplementary Figure 1b). The ancilla and the cavities are each undercoupled to separate pins through which they are driven by optimal control pulses[4] to prepare the desired initial states. The ancilla coupling pin is also used for applying the sideband pump tone and for driving the readout resonator. In addition, the readout resonator is overcoupled to a pin that transmits the readout signal to a Josephson parametric converter (JPC), followed by a high electron mobility transistor (HEMT) amplifier, allowing single-shot readout of the ancilla state.

Supplementary Note 2: System Hamiltonian

The four modes of the device involved in the experiment can be approximately described up to fourth order in their fields by the Hamiltonian

$$\begin{aligned}\hat{H}_0/\hbar &= \omega_{\mathrm{C}}\hat{a}_{\mathrm{C}}^{\dagger}\hat{a}_{\mathrm{C}} + \omega_{\mathrm{T}}\hat{a}_{\mathrm{T}}^{\dagger}\hat{a}_{\mathrm{T}} + \omega_{\mathrm{r}}\hat{a}_{\mathrm{r}}^{\dagger}\hat{a}_{\mathrm{r}} + \omega_{ge}|e\rangle\langle e| + \omega_{gf}|f\rangle\langle f| \\ &\quad -\widetilde{\chi}_{\mathrm{C}}^{e}\hat{a}_{\mathrm{C}}^{\dagger}\hat{a}_{\mathrm{C}}|e\rangle\langle e| - \widetilde{\chi}_{\mathrm{T}}^{e}\hat{a}_{\mathrm{T}}^{\dagger}\hat{a}_{\mathrm{T}}|e\rangle\langle e| - \widetilde{\chi}_{\mathrm{r}}^{e}\hat{a}_{\mathrm{r}}^{\dagger}\hat{a}_{\mathrm{r}}|e\rangle\langle e| \\ &\quad -\widetilde{\chi}_{\mathrm{C}}\hat{a}_{\mathrm{C}}^{\dagger}\hat{a}_{\mathrm{C}}|f\rangle\langle f| - \widetilde{\chi}_{\mathrm{T}}\hat{a}_{\mathrm{T}}^{\dagger}\hat{a}_{\mathrm{T}}|f\rangle\langle f| - \widetilde{\chi}_{\mathrm{r}}\hat{a}_{\mathrm{r}}^{\dagger}\hat{a}_{\mathrm{r}}|f\rangle\langle f| \\ &\quad -\chi_{\mathrm{CT}}\hat{a}_{\mathrm{C}}^{\dagger}\hat{a}_{\mathrm{C}}\hat{a}_{\mathrm{T}}^{\dagger}\hat{a}_{\mathrm{T}} - \chi_{\mathrm{Cr}}\hat{a}_{\mathrm{C}}^{\dagger}\hat{a}_{\mathrm{C}}\hat{a}_{\mathrm{r}}^{\dagger}\hat{a}_{\mathrm{r}} - \chi_{\mathrm{Tr}}\hat{a}_{\mathrm{T}}^{\dagger}\hat{a}_{\mathrm{T}}\hat{a}_{\mathrm{r}}^{\dagger}\hat{a}_{\mathrm{r}} \\ &\quad -\frac{\chi_{\mathrm{CC}}}{2}\hat{a}_{\mathrm{C}}^{\dagger}\hat{a}_{\mathrm{C}}^{\dagger}\hat{a}_{\mathrm{C}}\hat{a}_{\mathrm{C}} - \frac{\chi_{\mathrm{TT}}}{2}\hat{a}_{\mathrm{T}}^{\dagger}\hat{a}_{\mathrm{T}}^{\dagger}\hat{a}_{\mathrm{T}}\hat{a}_{\mathrm{T}} - \frac{\chi_{\mathrm{rr}}}{2}\hat{a}_{\mathrm{r}}^{\dagger}\hat{a}_{\mathrm{r}}^{\dagger}\hat{a}_{\mathrm{r}}\hat{a}_{\mathrm{r}},\end{aligned} \tag{1}$$

where the readout mode is denoted by 'r'. The parameters in this Hamiltonian are specified in Supplementary Table 1, and the coherence properties of the modes are described in Supplementary Table 2. The first row in Supplementary Equation 1 describes the transition frequencies of the modes, and the second and third rows contain the dispersive interaction terms of the ancilla $|e\rangle$ and $|f\rangle$-states with the readout resonator and the cavities, which are all in the few megahertz range. The final two rows describe the cross-Kerr interaction terms between the readout and cavity modes, as well as their self-Kerr rates, which are all in the few kilohertz range. The only term in this free-evolution Hamiltonian that is explicitly used in the CNOT gate protocol is the dispersive interaction between the target cavity and the ancilla in the $|f\rangle$-state at a rate $\widetilde{\chi}_{\mathrm{T}}$, which also determines the ultimate speed limit of the gate.

Supplementary Table 1: Parameters of the full system Hamiltonian.

Term ($/2\pi$)	Measured (Predicted)	Term ($/2\pi$)	Measured (Predicted)
ω_{C}	4.22 GHz	ω_{ge}	4.79 GHz
ω_{T}	5.45 GHz	ω_{gf}	9.46 GHz
ω_{r}	7.70 GHz		
$\widetilde{\chi}_{\mathrm{C}}^{e}$	1.02 MHz	$\widetilde{\chi}_{\mathrm{C}}$	3.3 MHz
$\widetilde{\chi}_{\mathrm{T}}^{e}$	1.10 MHz	$\widetilde{\chi}_{\mathrm{T}}$	1.9 MHz
$\widetilde{\chi}_{\mathrm{r}}^{e}$	1.74 MHz	$\widetilde{\chi}_{\mathrm{r}}$	(3.3 MHz)
χ_{CT}	2 kHz	χ_{CC}	(1.6 kHz)
χ_{Cr}	(5 kHz)	χ_{TT}	(3.4 kHz)
χ_{Tr}	(12 kHz)	χ_{rr}	(7 kHz)

Supplementary Table 2: **Coherence properties.** Energy relaxation time (T_1), dephasing time (T_2^*), and thermal population (P_e) of the system components. The ancilla and cavity states are measured before each run of the experiment to verify the absence of thermal excitations.

	T_1	T_2^*	P_e
Control cavity:	∼2.2 ms	∼0.5 ms	2-3%
Target cavity:	∼2.0 ms	∼0.6 ms	2-3%
Readout resonator:	300 ns	N/A	<0.2%
Ancilla $\lvert e\rangle$:	60 μs	37±5 μs	7.5%
Ancilla $\lvert f\rangle$:	40 μs	17±5 μs	∼0.5%

Supplementary Note 3: Driven cavity-ancilla sideband interaction

The source of nonlinearity in our system is the Josephson junction of the ancilla transmon, whose Hamiltonian is[5]

$$\begin{aligned}\hat{H}_{\mathrm{J}} = -E_{\mathrm{J}}\cos\Big[&\phi_q\left(\hat{q}+\hat{q}^{\dagger}+\xi(t)+\xi^*(t)\right) \\ &+\phi_{\mathrm{C}}\left(\hat{a}_{\mathrm{C}}+\hat{a}_{\mathrm{C}}^{\dagger}\right)+\phi_{\mathrm{T}}\left(\hat{a}_{\mathrm{T}}+\hat{a}_{\mathrm{T}}^{\dagger}\right)+\phi_{\mathrm{r}}\left(\hat{a}_{\mathrm{r}}+\hat{a}_{\mathrm{r}}^{\dagger}\right)\Big],\end{aligned} \tag{2}$$

where $E_{\mathrm{J}}/h = 21$ GHz is the Josephson energy, and $\hat{q}$ is the ancilla mode annihilation operator. ϕ_k are the normalized zero point flux fluctuations across the junction due to mode k, and $\xi(t) \approx \frac{g(t)}{\Delta}$ is the displacement of the ancilla mode when driven by a pump tone at a rate $g(t)$ and detuning Δ. In the limit of small flux through the junction, this Hamiltonian can be approximated by the fourth order term in the expansion of the cosine. In this limit, the Josephson junction acts as a four-wave mixing element. When the junction is driven by a pump, terms that would otherwise be non-energy conserving can be accessed. In particular, the pumped four-wave mixing interaction

of interest to us is

$$\hat{H}_{\rm sb} = -\frac{1}{2} E_{\rm J} \phi_q^3 \phi_{\rm C} \xi(t) \left(\hat{a}_{\rm C} \hat{q}^\dagger \hat{q}^\dagger + \hat{a}_{\rm C}^\dagger \hat{q}\, \hat{q} \right), \tag{3}$$

which describes a sideband transition between the control cavity and the ancilla. Through this interaction a single pump photon is absorbed, while extracting a single photon from the control cavity and doubly exciting the ancilla (see Fig. 2b in the main text). This term can be made resonant provided the pump satisfies the frequency condition $\omega_{\rm p} = \omega_{gf} - \omega_{\rm C} - (n_{\rm C} - 1)\widetilde{\chi}_{\rm C}$, (we discuss the effect of $n_{\rm T}$ in the next section). This interaction is a natural choice for implementing the CNOT gate. This can be seen by noting that any pumped cavity-ancilla interaction needs to involve at least a single pump photon, a single cavity photon and a single ancilla excitation. However, the four-wave mixing interaction requires a fourth additional photon. Since the contribution of the ancilla to the junction's zero point flux fluctuation ($\phi_q = 0.32$) is stronger than that of the control cavity ($\phi_{\rm C} = 0.016$) or that of the pump (assuming $|\xi(t)| < 1$), a second ancilla excitation provides the fastest possible nonlinear interaction.

The fourth-order approximation of the cosine Hamiltonian is valid only for $\phi_q |\xi(t)| \ll 1$, setting a limit on how strongly we can pump the sideband interaction. When approaching this limit, the interaction strength will saturate, and higher-order spurious nonlinear processes are likely to appear as well. In order to calibrate the pump strength, we measure the Stark shift of the ancilla frequency (due to the term $-E_{\rm J}\phi_q^4 |\xi(t)|^2 \hat{q}^\dagger \hat{q}$). From this, we can infer a pump strength corresponding to $\phi_q \xi \sim 0.16$ (or $\xi \sim 0.5$). Supplementary Equation 3 then predicts an oscillation rate of $\sqrt{2}\Omega_{\rm C} \sim 2\pi \times 11$ MHz with two photons in the control cavity, in close agreement with the measured value.

Supplementary Note 4: Nonidealities in the gate protocol

In the discussion of the gate protocol in the main text, we ignored the effect of the dispersive ancilla-target interaction $\widetilde{\chi}_{\rm T} \hat{a}_{\rm T}^\dagger \hat{a}_{\rm T} |f\rangle\langle f|$ on the pumped ancilla-control sideband interaction. In reality, however, different photon numbers $n_{\rm T}$ in the target cavity translate into different pump frequency matching conditions $\omega_p = \omega_{gf} - \omega_{\rm C} - (n_{\rm C} - 1)\widetilde{\chi}_{\rm C} - n_{\rm T}\widetilde{\chi}_{\rm T}$. If we match the pump frequency for $n_{\rm T} = 2$, the sideband oscillations for $|0_{\rm T}\rangle$ and $|4_{\rm T}\rangle$ will have a contrast reduced by approximately $(2\widetilde{\chi}_{\rm T})^2/(\sqrt{2}\Omega_{\rm C})^2 \sim 10\%$. Therefore, we cannot excite the ancilla to $|f\rangle$ for all target states simultaneously (See Supplementary Figure 2a). At first sight this appears to be a limiting factor of the gate. However, complete excitation to $|f\rangle$ is not required for the CNOT gate, as long as the ancilla returns to the ground state by the end of the operation, and provided $|2_{\rm T}\rangle$ acquires a relative phase of π with respect to $|0_{\rm T}\rangle$ and $|4_{\rm T}\rangle$. Indeed, these requirements can be met by appropriate tuning of the gate parameters. To see this, consider first the case of $|2_{\rm C}, 2_{\rm T}, g\rangle$, for which the pump is on resonance. Regardless of the pump pulse duration $t_{\rm p}$ and the wait time $t_{\rm w}$, the area enclosed during the trajectory on the Bloch sphere composed by the two levels $|2_{\rm C}, 2_{\rm T}, g\rangle$ and $|2_{\rm C}, 1_{\rm T}, f\rangle$ (Supplementary Figure 2c) is zero (assuming that the initial and final pulse have opposite phases). The goal is then to make $|2_{\rm C}, 0_{\rm T}, g\rangle$ and $|2_{\rm C}, 4_{\rm T}, g\rangle$ acquire a total (geometric and dynamic) phase of π during a closed trajectory on the Bloch sphere. Since the sideband transitions are detuned from resonance by an equal amount $\pm 2\widetilde{\chi}_{\rm T}$ for both states, both will trace identical trajectories on the Bloch sphere, albeit in opposite directions. A closed trajectory can always be achieved by first fixing $t_{\rm p}$, and then choosing $t_{\rm w}$ such that the Bloch vector rotates to its mirror image with respect to the axis of rotation (see Figs. S2b-d). The second sideband pulse will then always bring the ancilla back to its ground state. Next, $t_{\rm p}$ can be varied together with $t_{\rm w}$ obtained by the above procedure, until a total phase of π is acquired. Due to a nonzero ratio $\widetilde{\chi}_{\rm T}/\Omega_{\rm C}$, the resulting wait time and pulse duration will deviate from the simplified expressions provided in the main text $t_{\rm w} = \pi/2\widetilde{\chi}_{\rm T} = 130$ ns and $t_{\rm p} = \pi/(\sqrt{2}\Omega_{\rm C}) = 45$ ns. Instead, a simulation gives values of $t_{\rm w}^{\rm sim} = 83$ ns and $t_{\rm p}^{\rm sim} = 49$ ns (Supplementary Figure 2b-d). However, in the actual experiment, $t_{\rm w} = 100$ ns and $t_{\rm p} = 45$ ns are found to satisfy the above requirements. This discrepancy is explained by a lowering of the ancilla-target dispersive interaction when the pump is switched on from $\widetilde{\chi}_{\rm T} = 2\pi \times 1.9$ MHz to $\widetilde{\chi}_{\rm T}^{\rm pump} = 2\pi \times 1.4$ MHz. For this procedure to work, it is important for both $n_{\rm T} = 4$ and $n_{\rm T} = 0$ to acquire the same total phase, which is ideally obtained by setting the pump frequency exactly on resonance for $n_{\rm T} = 2$. However, in the experiment, a $\sim 2\pi \times 1$ MHz deviation from this frequency, or a higher-order nonlinearity of the same magnitude, may be responsible for the relatively large gate infidelity whenever the target cavity state is not rotationally symmetric, as observed in Fig. 4b of the main text. Indeed, state tomography on those states confirms that the infidelity originates mainly from a relative phase between $n_{\rm T} = 0$ and $n_{\rm T} = 4$.

Single-qubit rotations are another effect that needs to be taken into account. In the case of the control qubit, the deterministic rotation acquired due to Stark shifts and dispersive interaction with the ancilla can be annulled by fixing the phase of the second sideband pump pulse accordingly. For the target qubit, the phase acquisition due to the Stark shift is independently measured and removed in post-processing of the data.

Supplementary Note 5: State Preparation And Measurement errors

As emphasized in the main text, quantum state tomography and quantum process tomography (QPT) are limited by imperfections in state preparation and measurement. An estimate of the minimal infidelity of the initial states is provided by the ratio of the control pulse duration to the ancilla dephasing time, given by 1 μs / 37 μs $\approx 3\%$. The multiple mechanisms leading to measurement errors are discussed in detail in Ref. [6].

Supplementary Note 6: Density matrix reconstruction

For reconstructing the density matrix $\hat{\rho}_{\rm CT}$ of the joint two-cavity system, we first measure its joint Wigner distribution. This is done by measuring the joint parity $\hat{P} = \exp\left[i\pi\left(\hat{a}_{\rm C}^\dagger \hat{a}_{\rm C} + \hat{a}_{\rm T}^\dagger \hat{a}_{\rm T}\right)\right]$ of the cavities[6] after displacing them in their four-dimensional phase space:

$$W_{\rm J}(\beta_{\rm C}, \beta_{\rm T}) = \frac{4}{\pi^2} {\rm Tr}\left[\hat{\rho}_{\rm CT} \hat{D}_{\beta_{\rm C}} \hat{D}_{\beta_{\rm T}} \hat{P} \hat{D}_{\beta_{\rm C}}^\dagger \hat{D}_{\beta_{\rm T}}^\dagger\right], \tag{4}$$

where $\hat{D}_\beta = e^{\beta \hat{a}_i^\dagger - \beta^* \hat{a}_i}$ is a displacement by β of the state of cavity $i = {\rm C}, {\rm T}$. Joint parity measurements are performed by a Ramsey interferometry measurement on the ancilla, which is subsequently read out. To compensate for imperfections in this procedure[6], we calibrate the parity measurements using the value obtained for the vacuum state (0.79 ± 0.02). If we assume cutoffs $N_{\rm C}$ and $N_{\rm T}$ of the photon numbers in the control and target cavities, we can write $\hat{\rho}_{\rm CT}$ as a $(N_{\rm C}N_{\rm T}) \times (N_{\rm C}N_{\rm T})$ matrix. By measuring the joint Wigner distribution at this number of displacements or more, we can perform a maximum

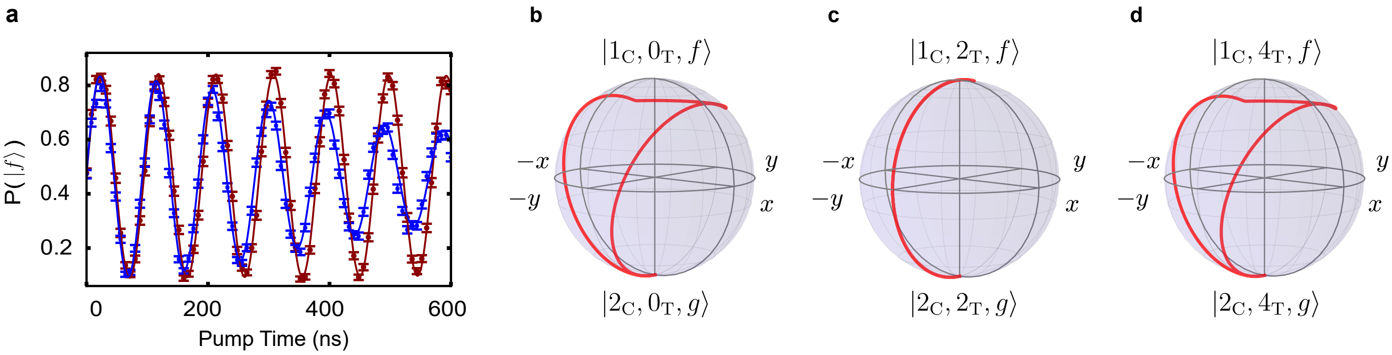

Supplementary Figure 2: **Effect of the target-ancilla dispersive interaction on the sideband transitions. a,** The sideband transition is on resonance when two photons are present in the target cavity (red curve). If the target cavity contains a kitten state instead, the dispersive interaction with the ancilla results in a gradual dephasing of the sideband oscillations (blue curve). Error bars indicate the standard error of the mean. **b, c, d,** Simulated Bloch sphere trajectories for the gate protocol (pump pulse - wait - pump pulse), with $n_{\rm T}$ = 0 (**b**), 2 (**c**) and 4 (**d**). In this simulation, $\widetilde{\chi}_{\rm T}/2\pi$ = 1.9 MHz and $\sqrt{2}\Omega_{\rm C}/2\pi$ = 11 MHz. By choosing a 49 ns pump pulse and a 83 ns wait time, we end up in the ground state regardless of $n_{\rm T}$, and acquire a phase of π for $n_{\rm T} = 0$ and $n_{\rm T} = 4$ with respect to $n_{\rm T} = 2$.

likelihood estimation to infer the most probable positive semi-definite Hermitian matrix $\hat{\rho}_{\rm CT}$. In practice, we use 6^4 different displacements, and reconstruct the two-cavity density matrix assuming fewer than six photons per cavity. We then confirm that up to the measurement accuracy there are at most four photons in the target cavity, and at most two photons in the control cavity for all measured states. This allows us then to reconstruct $\hat{\rho}_{\rm CT}$ for this restricted 15-dimensional Hilbert space, using a now overcomplete set of data.

Since the trace of the density matrix is not constrained to unity, this method does not make the a priori assumption that the gate operation is uncorrelated with tomography errors. Instead, failures of tomography as a result of the gate operation will show up as a reduced trace, and hence a reduced state fidelity of the final density matrix.

Supplementary Note 7: Encodings compatible with the CNOT gate

Single-photon encoding

The simplest possible encoding compatible with the gate protocol presented in the main text is the single-photon encoding, with $|0\rangle_{\rm C}(|0\rangle_{\rm T})$ and $|1\rangle_{\rm C}(|1\rangle_{\rm T})$ as the basis states for the control (target) cavity. While this encoding offers the longest possible qubit lifetimes, it cannot be used for either error detection or error correction. The gate protocol is identical to that of the multiphoton encoding, albeit with different timings. The presence of a single photon in the control cavity instead of two reduces the sideband oscillation rate by a factor of $\sqrt{2}$ due to the absence of bosonic enhancement. The pump tone is therefore applied for 64 ns instead of 45 ns. If the ancilla is excited to $|f\rangle$, the dispersive interaction between the target and the ancilla sets in. However, for the single-photon encoding, the rotation in the target cavity phase space required for turning $|0\rangle_{\rm T}+|1\rangle_{\rm T}$ into $|0\rangle_{\rm T}-|1\rangle_{\rm T}$ is π instead of $\pi/2$, making this step about twice as long. In total, the gate time is 340 ns instead of 190 ns for the multiphoton encoding. We perform QPT for the resulting gate (Supplementary Figure 3), and measure a process fidelity of $\rm F_{CNOT} = (95 \pm 2)\,\%$. As in the case of the multiphoton encoding, state preparation and measurement cannot be isolated from the gate operation. Indeed, when performing QPT on the process consisting of encoding and readout only, we observe a similar value for the process fidelity with the expected identity operator of $\rm F_{identity} = (98 \pm 1)\,\%$. We therefore conclude that the effect of gate errors on the gate fidelity is obscured by state preparation and measurement errors. The single-photon encoding is used for the initial states in Fig. 5 of the main text. The density matrices of these states are presented in Supplementary Figure 4.

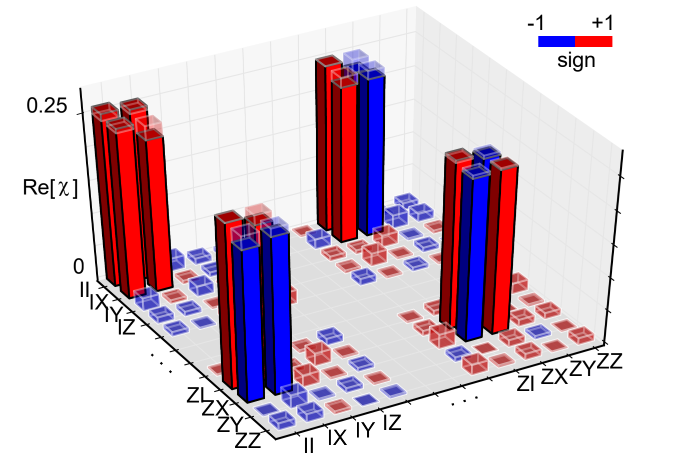

Supplementary Figure 3: **Quantum process tomography of the CNOT gate with single-photon encoding.** The solid (transparent) bars represent the measured (ideal) elements of the process matrix χ. The corresponding process fidelity is $\rm F_{CNOT} = (95 \pm 2)\,\%$. For clarity, only the corners of the process matrix are presented here.

The single-photon encoding, as well as the multiphoton encoding used in the main text are just two instances of a class of encodings that is compatible with the gate operation. In the control cavity, any encoding of the form $|0_{\rm L}\rangle_{\rm C} = |0\rangle_{\rm C}, |1_{\rm L}\rangle_{\rm C} = |n\rangle_{\rm C}$ can be used. For the target cavity, any two orthogonal states satisfying $|0_{\rm L}\rangle_{\rm T} = e^{\pm i\hat{a}_{\rm T}^{\dagger}\hat{a}_{\rm T}\theta}|1_{\rm L}\rangle_{\rm T}$ are compatible with the gate, since these states are interchanged by a phase space rotation. However, for this class of encodings a photon loss in the control cavity can only be detected, and not corrected. This is because the control cavity collapses to $|n-1\rangle_{\rm C}$ regardless of the initial state, thereby losing the stored information. Moreover, in the absence of a photon loss event, the state is gradually projected onto vacuum. This loss of information does not lead to a final state outside the code space, and is therefore an undetectable error mechanism.

Generalized kitten encoding

The multiphoton encodings discussed above are different for both qubits, and can enable full error correction only for the target qubit. Conventional error-correctable states such as the kitten states cannot be used for encoding the control qubit in the current scheme, since for the Fock states $|2\rangle$ and $|4\rangle$ the ratio of the sideband oscillation rates $\sqrt{n_{\mathrm{C}}}\Omega_{\mathrm{C}}$ is irrational.

However, we can introduce a generalized kitten encoding with basis states $|0_{\mathrm{L}}\rangle = \frac{1}{\sqrt{2}}\left(\frac{\sqrt{3}|0\rangle+|8\rangle}{2} + |2\rangle\right)$ and $|1_{\mathrm{L}}\rangle = \frac{1}{\sqrt{2}}\left(\frac{\sqrt{3}|0\rangle+|8\rangle}{2} - |2\rangle\right)$. These states have equal photon loss probability, and collapse onto orthogonal states when a single photon is lost. Therefore, this encoding satisfies the criteria for error-correctability[7]. In contrast to kitten states, these generalized kitten states are potentially compatible with the CNOT gate, and can be used to encode both the control and target qubits. A pump pulse that results in a full sideband transition $|2\rangle_{\mathrm{C}}|g\rangle \to |1\rangle_{\mathrm{C}}|f\rangle$ corresponds to a back and forth transition for $|8\rangle_{\mathrm{C}}|g\rangle$. As a result, only $|2_{\mathrm{C}}\rangle$ leads to an excitation of the ancilla to $|f\rangle$, and therefore to a rotation of the target cavity phase space, whereas $\frac{1}{2}(\sqrt{3}|0_{\mathrm{C}}\rangle + |8_{\mathrm{C}}\rangle)$ leaves the target qubit unchanged. In addition, this encoding does not entail a decrease in qubit lifetime as compared to the kitten encoding, since the average number of photons remains two. However, the presence of higher photon states requires a lower ratio $\widetilde{\chi}_{\mathrm{T(C)}}/\Omega_{\mathrm{C}}$ than that provided by the present experimental setup, and may also increase susceptibility to higher order terms that are not taken into account in this work.

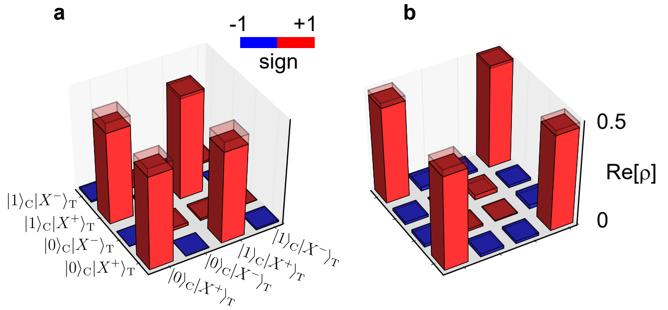

Supplementary Figure 4: **Entangled state generation in the single-photon encoding.** Real parts of the reconstructed density matrices (solid bars) of **a,** the initial separable two-cavity state $(|0\rangle_{\mathrm{C}} + |1\rangle_{\mathrm{C}}) \otimes (|0\rangle_{\mathrm{T}} + |1\rangle_{\mathrm{T}})$ (ideal shown in transparent bars), and **b,** the entangled state $|0\rangle_{\mathrm{C}}(|0\rangle_{\mathrm{T}} + |1\rangle_{\mathrm{T}}) + |1\rangle_{\mathrm{C}}(|0\rangle_{\mathrm{T}} - |1\rangle_{\mathrm{T}})$ after application of the CNOT gate. For clarity, the target state is shown in the basis $|X^{\pm}\rangle_{\mathrm{T}} = (|0\rangle_{\mathrm{T}} \pm |1\rangle_{\mathrm{T}})$.

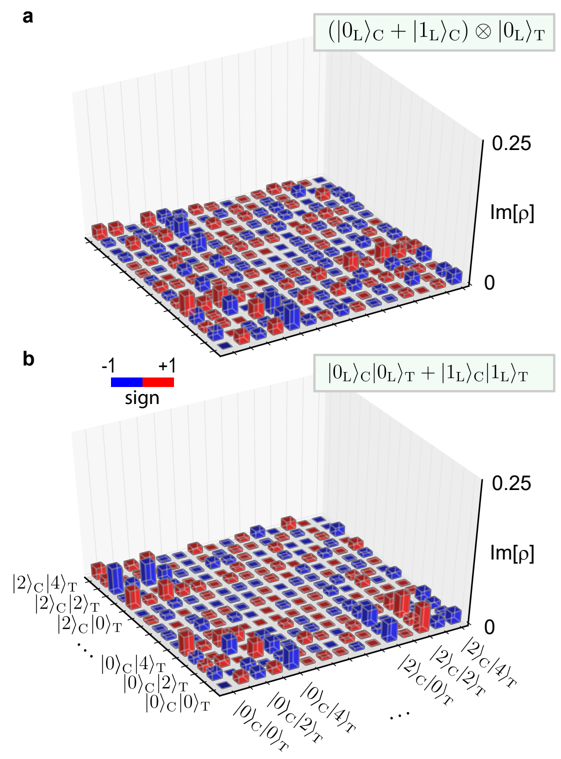

Supplementary Figure 5: **Generation of a multiphoton Bell state.** Imaginary parts of the reconstructed density matrices of **a,** the initial separable two-cavity state $(|0\rangle_{\mathrm{C}}+|2\rangle_{\mathrm{C}})\otimes(\frac{|0\rangle_{\mathrm{T}}+|4\rangle_{\mathrm{T}}}{\sqrt{2}}+|2\rangle_{\mathrm{T}})$ and **b,** the multiphoton Bell state after application of the CNOT gate. The real parts are shown in Fig. 3 of the main text.

Supplementary Note 8: Additional Data

In Supplementary Figure 5 we show the imaginary parts of the density matrices of the separable input state and the multiphoton Bell state obtained after application of the CNOT gate. The real parts are presented in Fig. 3 of the main text.

In Supplementary Figure 6, the full process matrix of the CNOT gate is presented. Only the corners of the real part are presented in Fig. 4a of the main text for clarity.

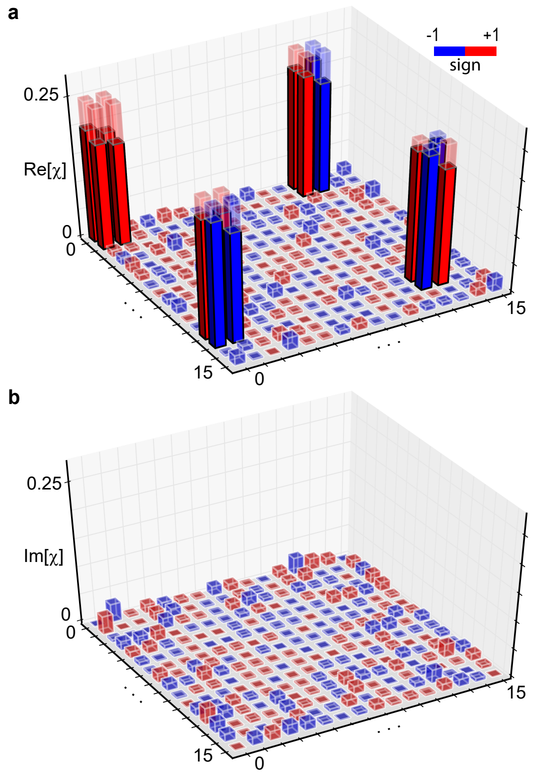

Supplementary Figure 6: **Quantum process tomography. a,** Real and **b,** imaginary part of the full process matrix χ for the multiphoton encoding. The solid (transparent) bars represent the measured (ideal) elements of the process matrix. The indices correspond to the two-qubit logical Pauli operators $\{\mathrm{I,X,Y,Z}\}^{\otimes 2} = \{\mathrm{II,IX,IY,...,ZZ}\}$.

Supplementary References

[1] Reagor, M. *et al.* Reaching 10 ms single photon lifetimes for superconducting aluminum cavities. *Appl. Phys. Lett.* **102**, 192604 (2013).

[2] Axline, C. *et al.* An architecture for integrating planar and 3d cQED devices. *Appl. Phys. Lett.* **109**, 042601 (2016).

[3] Reagor, M. *et al.* Quantum memory with millisecond coherence in circuit QED. *Phys. Rev. B* **94**, 014506 (2016).

[4] Heeres, R. W. *et al.* Implementing a universal gate set on a logical qubit encoded in an oscillator. *Nat. Commun.* **8**, 94 (2017).

[5] Leghtas, Z. *et al.* Confining the state of light to a quantum manifold by engineered two-photon loss. *Science* **347**, 853–857 (2015).

[6] Wang, C. *et al.* A Schrodinger cat living in two boxes. *Science* **352**, 1087–1091 (2016).

[7] Michael, M. H. *et al.* New class of quantum error-correcting codes for a bosonic mode. *Phys. Rev. X* **6**, 031006 (2016).
